# Sexual abuse among Mozambican women at risk for HIV/AIDS infection: The temporal stability of self‐report

**DOI:** 10.1002/jts.23178

**Published:** 2025-06-15

**Authors:** Ana Luísa Patrão, Teresa M. McIntyre, Eleonora C. V. Costa, Ângela Maia, Eduardo Matediana, Vanessa Azevedo

**Affiliations:** ^1^ Center for Psychology at University of Porto (CPUP), Faculty of Psychology and Education Sciences of the University of Porto University of Porto Porto Portugal; ^2^ Institute of Collective Health Federal University of Bahia Salvador Brazil; ^3^ Andy and Barbara Gessner College of Nursing and Texas Institute for Measurement, Evaluation and Statistics University of Houston Houston USA; ^4^ Department of Psychology, Centre for Philosophical and Humanistic Studies Portuguese Catholic University Braga Portugal; ^5^ Ministry of Health Lisbon Portugal; ^6^ School of Psychology University of Minho Braga Portugal; ^7^ Gynecology Department of the Central Hospital of Beira Beira Mozambique; ^8^ University of Maia Maia Portugal; ^9^ Observatory Permanent Violence and Crime (OPVC) University Fernando Pessoa Porto Portugal

## Abstract

Many researchers have expressed concern regarding the reliability and validity of retrospective self‐reports of sexual abuse. This study aimed to quantify the frequency of self‐reported sexual abuse among vulnerable Mozambican women and evaluate the temporal stability of self‐report across assessments. Participants (*N* = 173) were patients at the gynecology outpatient clinic of a public central hospital in Mozambique who were referred for recruitment by gynecologists and completed measures of sexual abuse, assessed using six items from the National Women's Study survey. Women reported a frequency of sexual abuse ranging from 9.2% (third assessment) to 10.4% (initial assessment). Concerning the temporal stability of self‐reports, the percentage of agreement was above 90% for all sexual abuse items, and general sexual victimization achieved almost perfect kappa values, κs = .93–1.00. This work has implications for the promotion of sexual health and the prevention of violence.

Intimate partner violence (IPV), including sexual abuse, is a major public health problem worldwide (World Health Organization [WHO], 2014), particularly in Mozambique (Chaquisse et al., 2018; W. M. Miller, 2019). In a 2015 survey, the prevalence of lifetime physical and/or sexual IPV was 22%, and the prevalence of past–12‐month physical and/or sexual intimate partner violence was 16% among Mozambican women (Ministry of Health & National Institute of Statistics, 2018). Sexual abuse in particular is strongly associated with HIV/AIDS infection in Mozambican women and girls (Ministry of Gender, Child, and Social Action, 2016), who demonstrated an HIV prevalence rate of 14.4%, among the highest in the world, in 2020 (Joint United Nations Programme on HIV/AIDS [UNAIDS], 2022). Aside from the risk of direct infection, violence and trauma appear to increase unhealthy behaviors, such as HIV/AIDS‐risk behaviors (Casique & Furegato, 2006; Gonçalves & Maia, 2021; Tarakeshwar et al., 2006; UNAIDS, 2004; Walsh et al., 2012; Wilson et al., 2012).

Both sexual abuse against women and HIV/AIDS infection can be related directly to a forced relationship with an infected person and related indirectly to risk behaviors, such as a lack of protection during sexual intercourse, drug abuse, having multiple sexual partners, the inability to negotiate condom use, and early sexual initiation with older men (Hassen & Deyassa, 2013; Seeley et al., 2004; Stockman et al., 2013; United Nations Population Fund, 2002). According to M. Miller's model (1999), this indirect association between sexual abuse and HIV/AIDS risk is regulated by three channels: (a) psychopathology as a consequence of trauma (e.g., depression, posttraumatic stress disorder), (b) drug use as a way to deal with the negativity resulting from the experience of sexual abuse, and (c) sexual adjustment problems related to decision‐making. These pathways, which mediate the association between sexual abuse and HIV/AIDS risk, are guided by mechanisms that drive AIDS‐risk behaviors, such as the use of drugs as self‐medication, self‐destructive behavior, and dissociation as a posttraumatic reaction. According to this model, abused women engage in risky sexual practices due to maladaptive strategies and beliefs (e.g., poor self‐esteem, poor perceived self‐efficacy, a sense of inability to protect themselves, hopelessness, difficulties in decision‐making, a sense of devaluation, intrusive thoughts, dissociation or cognitive distortions) that may appear independently as a reaction or in a complex scenario of psychopathological disorder.

In the context of female sexual health promotion—especially among the most vulnerable women, such as those in sub‐Saharan Africa, including Mozambique—it is important to examine in depth the impact of sexual abuse on the risk of HIV/AIDS. However, there are still concerns about the reliability and validity of retrospective self‐reports of sexual abuse, as many researchers have argued (e.g., Goldberg & Freyd, 2006; Hart & Rutter, 2004; Hepp et al., 2006; Krinsley et al., 2003; Spinhoven et al., 2012). Briefly, according to Dube et al. (2004), reliability indicates that self‐report is stable across time, whereas validity assesses its veracity. As a result, self‐report can be stable but not valid, whereas a valid report is necessarily stable. Research about these methodological concerns has focused mainly on reliability, especially temporal stability, as validity is constrained by the (im)possibility of the “truth” verification; therefore, it is beyond the scope of this research. Despite methodological concerns, usually presented as a limitation of empirical studies, research on the temporal stability of assessing sexual abuse by self‐report is still scarce (Gibbs et al., 2019; Loxton et al., 2019; Rowlands et al., 2021), with most research focusing on child sexual abuse (e.g., Langeland et al., 2014; Wielaard et al., 2018).

Most studies on sexual violence experiences of both children and adults have relied on retrospective self‐reports. Additionally, when studying the sexual violence victimization and its impact, studies have divided participants into two exposure groups based on self‐report: survivors (i.e., individuals who have self‐reported being survivors of sexual violence) and individuals who did not experience victimization (i.e., those who self‐reported not being survivors of sexual violence). Concerns about the temporal stability of these self‐reported experiences arise, as available evidence suggests that at least some participants change their answers when they are asked twice about the same experience. For instance, in a study by Fergusson et al. (2000), the authors concluded that there was poor agreement between reports of any child sexual abuse assessed at two time points (i.e., 18 and 21 years old) in the same sample, and using latent class analysis confirmed substantial measurement error with a high rate of false negative cases (i.e., individuals who survived sexual abuse failed to report the experience when asked about it). The reasons for change in self‐report are varied. For instance, Azevedo et al. (2021) conducted a cluster analysis and found that changes in self‐report could be explained by variables such as the valence, importance, and/or severity of the experience; developmental stage of occurrence; time interval between assessments; mode of assessment; interviewer characteristics; change of interviewer; mood; personality characteristics; physical and mental health; substance use; memory; secrecy; shame; third‐party protection; and help‐seeking. These studies suggest that the temporal stability of self‐reports of sexual abuse is an important area for further research. From a clinical perspective, and considering that in some settings, clients are prescreened before being allocated to a specific psychologist (e.g., a provider with a background in clinical psychology if a sexual violence experience is reported, a provider with a health psychology background when somatic issues and adaptation to disease are the critical issues, a provider with an educational psychology background if learning difficulties are identified), it is also relevant to understand whether self‐reports change over time to clarify if is necessary to ask more than once about those experiences throughout the psychological intervention. Repeated assessment may also be a clinical necessity in the case of a suspicion of underreporting.

In a study developed by Gibbs et al. (2019), the temporal stability of self‐reported IPV was assessed in a sample of 112 young women participants from South Africa, and the data were collected twice, with an interval of 2 weeks. The authors found that the prevalence of sexual IPV in the past year was 21.4% at Time (T) 1 and 15.2% at T2, and the lifetime prevalence was 40.2% at T1 and 42.0% at T2. Concerning the temporal stability of self‐reports, based on benchmarks outlined by Landis and Koch (1977), kappa values were moderate for both past‐year (κ = .44) and lifetime IPV sexual abuse experiences (κ = .56). In another recent study that examined the temporal stability of reporting IPV, including sexual abuse, Rowlands et al. (2021) analyzed 6,940 women from an Australian community sample on two different occasions, 12 months apart, who completed an online survey. The authors found that 89.1% of participants were consistent reporters (26% reported IPV, and the remaining reported no IPV, on both occasions), whereas 10.9% provided unstable reports, namely a history of IPV at the first assessment but not the second assessment. Moreover, the researchers also compared consistent and inconsistent reporters on a set of health variables and concluded that unstable reporters were less likely to report psychological distress, self‐harm, illicit drug use, suicidal ideation, self‐harm, and smoking. Despite its relevance, a limitation is that this study did not analyze sexual abuse independently from other types of abuse. However, these results seem to suggest more temporal stability of self‐report than other studies, at least regarding IPV.

This study aimed to fill a gap in the current literature on the temporal stability of self‐reports of sexual abuse among women at risk for HIV/AIDS infection. The literature on this topic has been scarce and has produced conflicting findings. This topic has not been studied in Mozambique, a country where the risk for HIV/AIDS is high, and studying the consistency of self‐reports of sexual abuse is relevant for intervention efforts. More specifically, we aimed to quantify the frequency of self‐reported sexual abuse at four different time points (T1–T4) and examine temporal stability over these four assessments, which were conducted over a period of approximately 3.5 months, in a sample of Mozambican women.

## METHOD

### Participants

Study participants at baseline (Time [T] 1) constituted a convenience sample of 173 Mozambican women at risk for sexual HIV/AIDS infection enrolled in an intervention program developed to promote sexual health. The study retained 164 women at T2 (postintervention), 156 women at T3 (1‐month follow‐up), and 150 women at T4 (3‐month follow‐up). The mean participant age at baseline was 24.7 years (*SD* = 5.5).

Participants were selected by gynecologists at the gynecology outpatient clinic of a public hospital in a large Mozambican city. Inclusion criteria were: (a) seeking medical care for sexually transmitted infections (STIs), (b) having had multiple sexual partners during the previous 6 months, (c) reporting having had sex with someone who was known to have had other sexual partners, or (d) having used injectable drugs. Gynecologists applied these criteria during gynecology visits. Patients were then referred to the research team, who followed the ethical procedures and applied the survey.

As shown in Table 1, the sample was composed of women between the 18 and 46 years of age, most of whom were single (41.6%) and had completed 7–9 (21.4%) or 10–12 (45.7%) years of education. Most of the women reported currently having a single partner (91.9%), previously having at least one STI (67.1%), having been tested for HIV/AIDS (84.4%), and not using condoms as a method of contraception (61.8%). It is important to note that 11.0% of women were already HIV positive and at risk for reinfection.

**TABLE 1:** Baseline demographic and sexual health characteristics of the sample.

Variable	*n*	%
Age (years)^a^		
≤ 20	42	24.3
21–25	75	43.4
> 25	56	32.4
Educational attainment		
4–6 years	41	23.7
7–9 years	37	21.4
10–12 years	79	45.7
Technical school	7	4.0
College (3–5 years)	9	5.2
Marital status		
Married	40	23.1
Common law	46	26.6
Single	72	41.6
Divorced	10	5.8
Widowed	5	2.9
Current partner is only partner		
Yes	159	91.9
No	14	8.1
Lifetime STI		
Yes	116	67.1
No	57	32.9
HIV test		
Yes	146	84.4
No	27	15.6
Lifetime HIV+		
Yes	19	11.0
No	154	89.0
Condom use as contraception		
Yes	66	38.2
No	107	61.8

*Note*: *N =* 173. STI = sexually transmitted infection.

^a^ *M*_age_ = 24.7 years, *SD* = 5.5, range: 18–46 years.

### Procedure

This study was part of a larger body of research conducted over 12 months that aimed to evaluate the effectiveness of two psychosocial interventions in preventing HIV/AIDS. Using a longitudinal approach, a 3‐month randomized controlled trial with three groups (two experimental, one control) was conducted. The samples for the larger study and the current study comprised the same women. This research had four evaluation moments: pretest (immediately before the intervention [T1]), posttest (immediately after the intervention and approximately 2 weeks after pretest [T2]), Follow‐Up 1 (1 month after the end of the intervention [T3]), and Follow‐Up 2 (3 months after the end of the intervention [T4]). Assessments took 40 min to complete. The results of the larger study and its methodology have been previously published (Patrão et al., 2024, 2021).

Data collection began after the study was approved by the Mozambican Minister of Health (Reference 151/020/GMS/09). This authorization process was carried out in person at the Mozambican Ministry of Health approximately 8 months before the study began. Women who agreed to participate in the study were given an informed consent form to read and sign. Participants were also informed that travel expenses (i.e., public transportation) to the hospital would be paid; no other financial incentives were offered.

### Measures

Sexual abuse that occurred after 16 years of age was assessed using six items from a questionnaire used in the National Women's Study (Resnick et al., 1993). The measure was initially part of the Women's Health Study Questionnaire (Hobfoll, 2002), and in the current study, we applied the Portuguese experimental version (McIntyre & Costa, 2004), adapted to the Mozambican context. Briefly, we used four items for rape, one item for sexual harassment, and one item for attempted sexual threats that occurred after 16 years of age. For each item, participants were asked about the occurrence (i.e., “yes” or “no”), the most recent experience (i.e., “last week,” “last month,” “1–3 months ago,” “3–6 months ago,” “6 months–2 years ago,” “2–6 years ago,” or “6 or more years ago”), and the total number of experiences. Items encompassed forced sex (“Made you have sex by using force or threatening to harm you or someone close to you?”), forced oral sex (“Made you have oral sex using force or threat of harm?”), forced anal sex (“Made you have anal sex using force or threat of harm?”), forced insertion of fingers and objects (“Put fingers or objects into your vagina or anus against your will by using force or threats?”), forced intimate touches (“Has anyone ever touched your breast, vagina, or anus, or made you touch his penis by using force or threat of harm?”), and any other type of forced sexual contact (“Have there been any other situation that did not involve sexual contact between you and another person but did involve an attempt by someone to force you to have any kind of unwanted sexual contact?)”

For the current study, only the occurrence (i.e., endorsement) of each item was analyzed. Based on these responses, we computed the overall number of sexual abuse experiences (0–6), which was recoded to distinguish nonexposed women (i.e., those who did not report any experiences) from survivors (i.e., those who reported at least one experience of sexual abuse). As these items were part of a survey and not rated on a standardized scale, no internal consistency reliability coefficient was computed. Thus, the six items were nested in a formative model (Bollen & Bauldry, 2011) instead of a reflective model (e.g., Coltman et al., 2008), which implies that categorization should be conceptual, the items are not interchangeable, and the items are independent from each other (i.e., being a survivor of one type of abuse does not imply being a survivor of a different type of abuse), as well as the absence of assumptions regarding item intercorrelation. Psychometric properties were not reported in the original studies, likely due to this reasoning.

### Data analysis

According to our study aims, univariate descriptive statistics were computed for the frequency of sexual abuse experiences over time. Based on the six original variables, we computed a new variable to identify survivors (i.e., participants who reported experiencing at least one kind of sexual abuse) and nonabused participants (i.e., did not report any experiences).

To explore the temporal stability of self‐reports, two distinct parameters of agreement were computed for each experience, as suggested by Sim and Wright (2005) and Kottner et al. (2011). Thus, the percentage of agreement, Cohen's kappa, and associated standard errors (Cohen, 1960; Fleiss et al., 2003) were analyzed for each individual item. Kappa values can range from 0 to 1.0 and to interpret the values, Landis and Koch's (1977) benchmarks were followed (i.e., poor: κ < .00, slight: .00–.20, fair: .21–.40, moderate: .41–.60, substantial: .61–.80, and almost perfect: .81–1.00. It should be noted that in some circumstances (i.e., when all values correspond to a single cell or are distributed between only two cells), kappa cannot be computed because variables are constant.

Data were analyzed using Excel and SPSS (Version 27) for Windows. As a longitudinal study, we considered that T1 represented a response rate of 100.0%; the response rate decreased to 94.8% at T2, 90.2% at T3, and 86.7% and T4. Missing values at each time point were assumed to be due to dropout, and no method was applied to replace them.

## RESULTS

### Frequency of sexual abuse

At all assessments, the most frequently reported experience of violence was forced sex, followed by forced oral sex and forced touches. See Table 2 for the frequencies of all reported sexual abuse types.

**TABLE 2:** Frequency of sexual abuse among Mozambican women across assessments

	Time 1	Time 2	Time 3	Time 4
Since you were 16 years old, have you had any of these experiences?	(*N* = 173)	(*n* = 164)	(*n* = 156)	(*n* = 150)
	**%**	**%**	**%**	**%**
Forced sex	9.8	9.8	10.3	9.4
Forced oral sex	1.2	1.2	0.6	1.3
Forced anal sex	0.6	1.2	0.6	0.7
Forced insertion of fingers and objects	0.6	1.2	0.0	0.7
Forced intimate touches	1.2	1.2	0.0	0.7
Any other type of forced sexual contact	0.6	0.6	0.0	0.0
Global sexual abuse^a^	10.4	10.3	9.2	9.7

^a^ Time 1: *n* = 18, Time 2: *n* = 16; Time 3: *n* = 16, Time 4: *n* = 15.

### Temporal stability of self‐reports of sexual abuse

As shown in Table 3, comparing assessments at T1 and T2, kappa values ranged from .49 (moderate) to 1.0 (almost perfect), and agreement was above 90% for all items. It should be noted that one item, “Have there been any other situation that did not involve sexual contact between you and another person but did involve an attempt by someone to force you to have any kind of unwanted sexual contact?,” presented a kappa value equal to 0 and an agreement of nearly 99%.

**TABLE 3:** Relative frequencies and agreement parameters on sexual abuse items across assessments

Types of behavior	Since you were 16 years old, have you had any of these forced experiences?															
	T1‐ T2				T2‐T3				T3‐T4				T1‐T4			
	Y/Y	N/N	κ	*SE*	Y/Y	N/N	κ	*SE*	Y/Y	N/N	κ	*SE*	Y/Y	N/N	κ	*SE*
	(%)	(%)			(%)	(%)			(%)	(%)			(%)	(%)		
Sex	9.8	90.2	1.0	.00	10.3	89.7	1	.00	9.4	89.9	.96	.04	9.4	89.9	.96	.04
Oral sex	0.6	98.2	.49	.31	0.6	98.7	.66	.32	0.7	98.7	.66	.32	0.7	98.0	.49	.31
Anal sex	0.6	98.8	.66	.32	0.6	98.7	.66	.32	0.7	99.3	1.0	.00	0.7	99.3	1.0	.00
Insertion of fingers and objects	0.6	98.0	.66	.32	0.0	98.7			0.0	99.3			0.7	99.3	1.0	.00
Intimate touches	0.6	98.2	.49	.31	0.0	98.7			0.0	99.3			0.7	98.7	.66	.32
Other sexual contact	0	98.8	0.0	.00	0.0	99.4			0.0	100			0.0	99.3		
Global sexual abuse	10.4	89.6	1.0	.00	10.3	89.1	.97	.03	9.4	89.3	.93	.05	9.7	89.0	.93	.05

*Note*: For empty cells, statistics were not computed because variables were constant, and crosstabs were empty or included a substantial proportion of zeros. T = Time; Y/Y = “yes” responses at both assessments; N/N = “no” responses at both assessments.

When we compared the T2 and T3 assessments, kappa values ranged from .66 (substantial) to 1.0 (almost perfect), with agreement of nearly 100%. For some items, kappa values could not be computed, as there was no variability in responses. A similar pattern was observed concerning T3 and T4. Finally, when comparing the first (T1) and last (T4) assessments, kappa values ranged from .49 (moderate) to 1.0 (almost perfect), with agreement of nearly 100%.

It should be noted that among participants who completed all four assessments, only 3.3% (*n* = 5) provided unstable self‐reports. Moreover, these participants provided 22 cases of unstable reporting; 63.6% of these unstable reports were changes from “yes” to “no,” and the remaining 36.4% were changes from “no” to “yes.” Among individuals who reported sexual violence at one assessment who had not reported exposure at a previous assessment, 75.0% said they preferred not to indicate the last time it happened, and the remaining 25.0% mentioned it happened in the previous 2–6 years.

## DISCUSSION

Most studies focused on the association between sexual abuse and risk for HIV/AIDS are based on retrospective self‐report, often using a cross‐sectional design. Besides analyzing the frequency of sexual abuse at four time points over a relatively lengthy period, this study addressed a well‐known concern, still scarcely investigated, especially in African women at risk for HIV/AIDS—namely, the temporal stability of self‐reports of sexual abuse that occurred after 16 years of age. Our findings show that the frequency of sexual abuse varied across the four assessments, with the highest frequency of reports found at T1 and the lowest at T4. The most prevalent sexual experience that participants reported was forced sex. The temporal stability of reports of sexual abuse was almost perfect, and the percentages of agreement were very high. Moreover, for individual items, kappa values ranged from moderate to almost perfect, and the percentages of agreement were consistently high.

Due to a lack of research among African women in general and Mozambican women in particular, it is difficult to compare our findings with other empirical studies aside from epidemiological investigations. However, many differences exist between these two types of studies, such as the sample composition. To our knowledge, there are no disaggregated data on the frequency of sexual abuse among Mozambican women, and although our values seem reasonable when compared to official data on IPV in this population (Ministry of Health & National Institute of Statistics, 2018), it should be mentioned that we did not limit reports of sexual violence experiences to those perpetrated by an intimate partner. At an international level, much more is known about child sexual abuse than about sexual abuse that occurs after 16 years of age, but according to a WHO report, 31% of women aged 15–49 have been subjected to physical and/or sexual violence perpetrated by a current or former husband or intimate partner, sexual violence perpetrated by a nonpartner, or both in their lifetime (WHO, 2021). Further, the report demonstrated a marked difference between higher‐income countries and low‐ and middle‐income countries, such as the case of Mozambique, and in the African region, the prevalence estimate of lifetime nonpartner sexual violence was reported to be 5%. Despite a lower rate than reported by Gibbs et al. (2019), the frequency of sexual abuse in our sample was approximately 10%, including partner and nonpartner violence, suggesting that Mozambican women are vulnerable to this kind of abuse, and this is an issue that should be addressed by health professionals, police, and politicians. Moreover, it seems reasonable to suspect that the frequency of sexual abuse may be even higher, as this type of experience is often underreported due to social desirability bias, memory phenomena, feelings of shame or guilt, or even as an effort to protect the offender (Hardt & Rutter, 2004; Krebs et al., 2022; Krumpal, 2013).

Although researchers have expressed concern regarding the temporal stability of self‐reported sexual abuse (e.g., Kendall‐Tackett & Becker‐Blease, 2004; Langeland et al., 2014), few studies have focused on this, and even fewer have included more than two assessment time points. According to our findings, self‐reports of sexual abuse remained quite stable over time, not only for individual experiences but also for the self‐identification of survivors and nonabused women. Indeed, a very small number of participants changed their reports concerning sexual abuse experiences over time. Again, the temporal stability of self‐reports was higher than what has been found by other researchers, such as Gibbs et al. (2019) or Rowlands et al. (2021), although caution should be taken in these comparisons due to some conceptual and methodological differences. Several factors may explain the values found in our study, including some design features, such as the short time intervals across assessments, or the behavioral nature of our questions. Other variables, such as familiarity with the researcher responsible for data collection, perpetrator anonymity (i.e., we asked about “a man” in general), or the minimization of memory effects due to the focus on experiences that occurred after 16 years of age, may have also contributed. Due to the low number of unstable reporters, it is difficult to identify a clear pattern of under‐ or overreporting. Underreporting appeared to be more frequent, which is similar to findings by Loxton et al. (2019, 2017), but distinct from the trend of overreporting found by Azevedo et al. (2022). Overreporting can occur due to the “reminiscence effect” or feeling more comfortable disclosing personal information, or it can result from new experiences. Rowlands et al. (2021) posited that inconsistency with regard to underreporting may be a sign of a resilient symptom trajectory, based on the reevaluation of past experiences and their personal meaning, and an attempt to provide “positive” narratives of one's life. Considering that women our sample were at high risk for HIV/AIDS and that they attended a psychological intervention devoted to sexual health promotion that achieved significant benefits (Patrão et al., 2021), linking inconsistency toward underreporting to a resilient trajectory maybe a reasonable explanation, although a more in‐depth analysis should be performed to empirically clarify this hypothesis. In fact, one aspect that may have contributed to temporal stability in this sample is the fact that these women were undergoing a psychological intervention that promoted their sexual health and confidence. The personal growth aspect of the program may have contributed to participants’ continued “ownership” of their adverse experiences via self‐reporting.

A comment should be made concerning the potential for the “kappa paradox,” which was first described by Feinstein and Ciccheti (1990) as a pitfall for researchers computing kappa statistics, especially in the field of life experiences (Hardt & Rutter, 2004). Intuitively, we would expect that sexual experiences with a high percentage of agreement would present high kappa values; however, this was not always the case, such as for the item “A man made you have oral sex using force or threat of harm.” There are two potential reasons to explain this paradox, namely, low frequencies and marginal distribution. To our knowledge, there is no option to replace kappa statistics on dichotomous variables; therefore, as many authors have recommended (e.g., Fleiss et al., 2003; Kottner et al., 2011; Sim & Wright, 2005), we presented several parameters (i.e., percentage of agreement, kappa statistics, and standard error) to allow for a more complete understanding of our results.

This study has some limitations that should be discussed. First, the findings are based on a convenience sample, namely women at risk for HIV/AIDS infection; thus, these participants are not representative of the general population, which limits the generalizability of the findings. Another potential limitation is the type of information collected regarding sexual abuse experiences. For instance, participants were not asked for any information about the perpetrator or the context of the abuse. We focused merely on the temporal stability of self‐reports, not on their validity. Therefore, we did not obtain independent corroboration of sexual abuse experiences. However, corroboration or collecting physical evidence of sexual violence, such as forced touches—one of the most frequently reported experiences in our sample—is almost impossible in most cases. In addition, we cannot fully guarantee that changes from “no” to “yes” did not represent new exposure that occurred since the previous assessment rather than inconsistency in reporting. Indeed, some participants in our sample preferred not to provide information about the time frame of the abuse, or when they did, it was not clear whether they were reporting a new incident. Lastly, as mentioned, several variables may underlie unstable reports, and we did not include any of these variables in the current study; consequently, these variables should be included in future studies to deepen the understanding of the findings. Given the high stability of reporting in this sample, it would be difficult to study unstable reporting in the current sample. Improving knowledge about the variables involved in reporting sexual abuse would benefit intervention efforts.

This study has several strengths, including its focus on a relevant and understudied topic of research. First, we focused on sexual abuse that occurred after 16 years of age, allowing for a better understanding of adolescent and adult survivors. Second, it increases the scope of current knowledge, traditionally restricted to abuse related to intimate partner relationships. Third, previous investigations of the temporal stability of self‐reports have relied on two assessment points, whereas in this study, we included four different assessment points. Finally, available research has focused on “WEIRD” samples, reflecting a term coined by Henrich et al. (2010) that refers to Western, educated, industrialized, rich, and democratic samples, and, to our best knowledge, this is one of the first studies based on an African sample.

Our work has implications for both research and clinical purposes. Overall, our results suggest that sexual abuse reports of Mozambican women at risk for HIV/AIDS tended to be stable. This finding indicates that clinicians and researchers working with this population can be reasonably confident about most of these women's self‐reports of sexual abuse. However, in this sample, a small number of reports were inconsistent, indicating that at least some reports will be inconsistent. Although it would be tempting to ignore them, a flexible but cautious approach is recommended. For instance, in clinical settings, psychologists should be flexible and open to changes in self‐reporting of sexual abuse over time. Researchers who ask mainly about occurrence on a “yes” or “no” dichotomous scale might also consider being more flexible in the answers provided, including measures of uncertainty or unwillingness to respond. It is important for clinicians and researchers to be mindful of potential factors in reporting so they can better understand the individual and sociocultural contexts of self‐reported sexual abuse.

## AUTHOR NOTES

This work was supported by a grant from the Portuguese Foundation for Science and Technology (SFRH/BD/37909/2007) and national funding from the Portuguese Foundation for Science and Technology, through the Center for Psychology at the University of Porto (CPUP; UIDB/00050/2020). This work was partially conducted at CIPsi, School of Psychology, University of Minho, supported by the Portuguese Foundation for Science and Technology (FCT; UID/01662: Centro de Investigação em Psicologia).

The authors gratefully acknowledge the contributions of the gynecologists of Beira Central Hospital, Mozambique. The authors also thank all the women who agreed to participate in this study.

The Article Processing Charge for the publication of this research was funded by the Coordenação de Aperfeiçoamento de Pessoal de Nível Superior ‐ Brasil (CAPES) (ROR identifier: 00x0ma614).

## OPEN PRACTICES STATEMENT

The data that support the findings of this study are available from the corresponding author. The data are not publicly available due to privacy or ethical restrictions.
